# Properties of Neurons in External Globus Pallidus Can Support Optimal Action Selection

**DOI:** 10.1371/journal.pcbi.1005004

**Published:** 2016-07-07

**Authors:** Rafal Bogacz, Eduardo Martin Moraud, Azzedine Abdi, Peter J. Magill, Jérôme Baufreton

**Affiliations:** 1 Medical Research Council Brain Network Dynamics Unit, Department of Pharmacology, University of Oxford, Oxford, United Kingdom; 2 Nuffield Department of Clinical Neurosciences, University of Oxford, Oxford, United Kingdom; 3 Univ. Bordeaux, Institut des Maladies Neurodégénératives, UMR 5293, Bordeaux, France; 4 CNRS, Institut des Maladies Neurodégénératives, UMR 5293, Bordeaux, France; George Mason University, UNITED STATES

## Abstract

The external globus pallidus (GPe) is a key nucleus within basal ganglia circuits that are thought to be involved in action selection. A class of computational models assumes that, during action selection, the basal ganglia compute for all actions available in a given context the probabilities that they should be selected. These models suggest that a network of GPe and subthalamic nucleus (STN) neurons computes the normalization term in Bayes’ equation. In order to perform such computation, the GPe needs to send feedback to the STN equal to a particular function of the activity of STN neurons. However, the complex form of this function makes it unlikely that individual GPe neurons, or even a single GPe cell type, could compute it. Here, we demonstrate how this function could be computed within a network containing two types of GABAergic GPe projection neuron, so-called ‘prototypic’ and ‘arkypallidal’ neurons, that have different response properties *in vivo* and distinct connections. We compare our model predictions with the experimentally-reported connectivity and input-output functions (f-I curves) of the two populations of GPe neurons. We show that, together, these dichotomous cell types fulfil the requirements necessary to compute the function needed for optimal action selection. We conclude that, by virtue of their distinct response properties and connectivities, a network of arkypallidal and prototypic GPe neurons comprises a neural substrate capable of supporting the computation of the posterior probabilities of actions.

## Author Summary

Choosing an appropriate action as quickly and accurately as possible in a given situation is critical for the survival of animals and humans. One of the brain regions involved in action selection is a set of subcortical nuclei known as the basal ganglia. The importance of understanding information processing in the basal ganglia is further emphasised by the fact that their disturbed interactions in Parkinson’s disease results in profound difficulties in movement. Computational models have suggested how the basal ganglia could select actions in the fastest possible way for the required accuracy level. These models further predict that a part of basal ganglia, called the external globus pallidus (GPe), needs to calculate a particular function of its inputs. This paper proposes how this function could be computed in a mathematical model of a network within GPe. Furthermore, it shows that the experimentally observed connectivity and response properties of GPe neurons fulfil the requirements necessary to support optimal action selection. This suggests the GPe neurons have properties that allow them to contribute to optimal action selection in the whole basal ganglia.

## Introduction

A set of subcortical brain nuclei known as the basal ganglia are thought to be involved in action selection [1]. The external globus pallidus (GPe; sometimes referred to as simply the ‘globus pallidus’ in rodents) plays an important role within the basal ganglia, in part because it is an ‘integrative hub’ that is connected to all other nuclei in these circuits [2,3]. The function of the GPe within the basal ganglia has been conceptualized in many computational models [4–8]. A class of models [9–12] suggests that, during selection of the most appropriate action, cortico-basal ganglia circuits approximate a statistical procedure known as the Multihypothesis Sequential Probability Ratio Test (MSPRT) [13]. These models assume the basal ganglia continuously update the probabilities of different actions being appropriate given sensory signals, and that an action is initiated whenever its corresponding probability exceeds a threshold of confidence. Such a procedure for making decisions has been shown analytically to yield the fastest possible choices for a given accuracy level, when the accuracy level approaches 100% [14], and in simulations with lower accuracy, the MSPRT makes faster or equally fast choices compared to other decision algorithms [15]. For brevity, we will refer to this property as the ‘optimal action selection’.

The optimal action selection models [9–12] assume that the GPe together with the subthalamic nucleus (STN), another basal ganglia nucleus, compute the normalization term from the equation of Bayes’ theorem. This normalization ensures that the probabilities represented in the basal ganglia add up to 1 across all actions. Hence the normalization computed by the STN-GPe network mediates the competition between actions by ensuring that an action is only selected when there is high evidence for it relative to the other options (this normalization is also critical to implement the MSPRT procedure, where the actual probabilities are compared against the threshold; thus, to perform MSPRT, it is not sufficient to just know the relative probabilities, as proposed in other Bayesian models [16]). The optimal action selection models have predicted that, in order to compute the normalization, the GPe needs to send feedback to the STN that is proportional to a particular function of STN activity (we review this function in detail below). However, because of the complex form of this function, it is not clear whether GPe neurons could compute it, and thus whether the basal ganglia could approximate the optimal action selection. The goal of this paper is to refine the mapping of the Bayes’ equation on the basal ganglia anatomy by taking into account new insights into GPe cell types, and investigate whether the GPe could compute this function.

In previous models of action selection in basal ganglia, it has been widely assumed that GPe neurons are homogenous in form and function [4–6,9,11,12,17]. However, recent work shows that the GABAergic projection neurons of the GPe can be divided into two main cell types, namely arkypallidal neurons and prototypic neurons. These two cell types exhibit largely distinct firing rates and patterns *in vivo*, including divergent encoding of spontaneous movements [18] as well as selective temporal coupling with different phases of the oscillations present in the cortex of dopamine-intact and Parkinsonian rodents [19–21]. The physiological dichotomy is mirrored by a molecular dichotomy. Thus, arkypallidal neurons express the transcription factor forkhead box protein 2 (FoxP2), whereas prototypic neurons do not [18,19]. Conversely, most prototypic neurons express the calcium-binding protein parvalbumin (PV), whereas arkypallidal neurons do not [18–20]. Equally important, arkypallidal and prototypic neurons preferentially innervate distinct sets of basal ganglia neurons [19,20], which we review in more detail below. In summary, there is now compelling support for the idea that a dichotomous functional organization, as actioned by arkypallidal and prototypic neurons with specialized physiological, molecular, and structural properties, is fundamental to the operations of the GPe. In this paper, we extend this notion by examining how a GPe network composed of these two distinct types of neuron could compute the function required for optimal action selection.

In the next section, we review the optimal action selection model and its predictions concerning computations in GPe. In the subsequent section, we show that the observed connectivity of arkypallidal and prototypic neurons, as well as the relationships between firing rate and injected current (f-I curves) for the two populations of the GPe neurons, fulfil the requirements necessary to approximate optimal action selection. Finally, we discuss our results and consider future directions.

## Model

We first review the overall computation in the model, and then its implementation in the cortico-basal-ganglia circuit.

### Computations in the model

Let us first introduce a simple choice task, in the context of which we present the model. Consider a rat that has to press either a lever to the left or to the right on the basis of an auditory stimulus. On each trial, pressing only one of the levers will lead to the reward. The auditory stimulus consists of a sequence of short intervals during which a low- or high-pitched tone is presented, which provide probabilistic information on which lever is correct on a given trial. During trials in which pressing the left lever is rewarded, the low tone has 70% chance of occurring in each interval, while the high tone has only 30% probability of occurring. Conversely, on trials when pressing the right lever is rewarded, the low and high tones have 30% and 70% probabilities, respectively. Let us assume that the rat is well trained in this task. Please note that, in this hypothetical task, in order to maximize its reward, the rat needs to listen to the stimulus, accumulate information from successive beeps, and then only makes a choice (i.e. selects an action) once it reaches a certain level of confidence.

Let us denote different actions available in a given context by *A*_*k*_, thus in the above example, the rat has two potentially rewarded actions, *A*_1_ and *A*_2_, corresponding to pressing the left and the right lever, respectively. The model suggests that, during action selection, the cortico-basal-ganglia circuit is evaluating the probabilities of alternative actions being appropriate in a given context, which we denote by *P*(*A*_*k*_). Whenever any of the probabilities exceeds a threshold of confidence during the internalized process of action selection, the corresponding action is triggered.

In the model, the probabilities of actions *P*(*A*_*k*_) are updated on the basis of sensory input. For simplicity, let us assume that the time during the action selection process is divided into discrete intervals, and during each interval a sensory input *S* in presented. The sensory input *S* could be used to update the probabilities of action *P*(*A*_*k*_), because from past experience the animal could have learned how often *S* appeared on trials on which action *A*_*k*_ was rewarded. Let us denote this rate of occurrence by *P*(*S*|*A*_*k*_). Thus, for example, in the task described above, if the low tone is presented at the current time step, then *P*(*S*|*A*_1_) = 0.7 and *P*(*S*|*A*_2_) = 0.3. Bayes’ theorem (see Eq 1) describes how to update the probabilities of actions according to the sensory input:

$$P\left(A_{k}|S\right)=\frac{P\left(A_{k}\right)P\left(S|A_{k}\right)}{P(S)}$$ (1)

Bayes’ theorem says that in order to compute the updated or ‘posterior’ probability of action *P*(*A*_*k*_|*S*), one needs to multiply the previous or prior probability *P*(*A*_*k*_) by the learned probability of the sensory input *S* appearing on trials on which action *A*_*k*_ was correct, i.e. *P*(*S|A*_*k*_). For example, when the low tone is presented, then *P*(*A*_1_) is multiplied by 0.7 and *P*(*A*_2_) is multiplied by 0.3. Additionally, to ensure that the posterior probabilities add up to 1, these products are divided by a normalization term *P*(*S*) equal to the sum of the products across all actions:

$$P\left(S\right)=\sum_{k=1}^{N}P\left(A_{k}\right)P\left(S|A_{k}\right)$$ (2)

In Eq 2, *N* denotes the number of available actions. If for any action the posterior probability computed from Eq 1 exceeds a threshold of confidence, the corresponding action is chosen. Otherwise the integration of information continues and the posterior probability *P*(*A*_*k*_|*S*) from the current time interval becomes the prior *P*(*A*_*k*_) for the next one.

### Neural implementation

Eq 1 includes multiplication and division, which are not natural operations for neurons (as classical neural networks models rather assume that neurons add their inputs and potentially transform them through non-linear functions e.g. [22]), but this problem can be solved by taking the logarithm. Recall that the logarithm has the following properties: log *a·b* = log *a* + log *b*, and log *a/b* = log *a*–log *b*. Hence taking the logarithm of both sides of Eq 1 we get:

$$\log P\left(A_{k}|S\right)=\log P\left(A_{k}\right)+\log P\left(S|A_{k}\right)-\log P(S)$$ (3)

Thus, if in the context of neurons, they have firing rates proportional to the logarithms of probabilities, the update according to Bayes’ theorem can be performed just using addition and subtraction. The computation of the logarithm of the normalization term becomes only slightly more complex, as it needs to include nonlinear transformations:

$$\log P\left(S\right)=\log\sum_{k=1}^{N}\exp\left(\log P\left(A_{k}\right)+\log P\left(S|A_{k}\right)\right)$$ (4)

**Fig 1:**
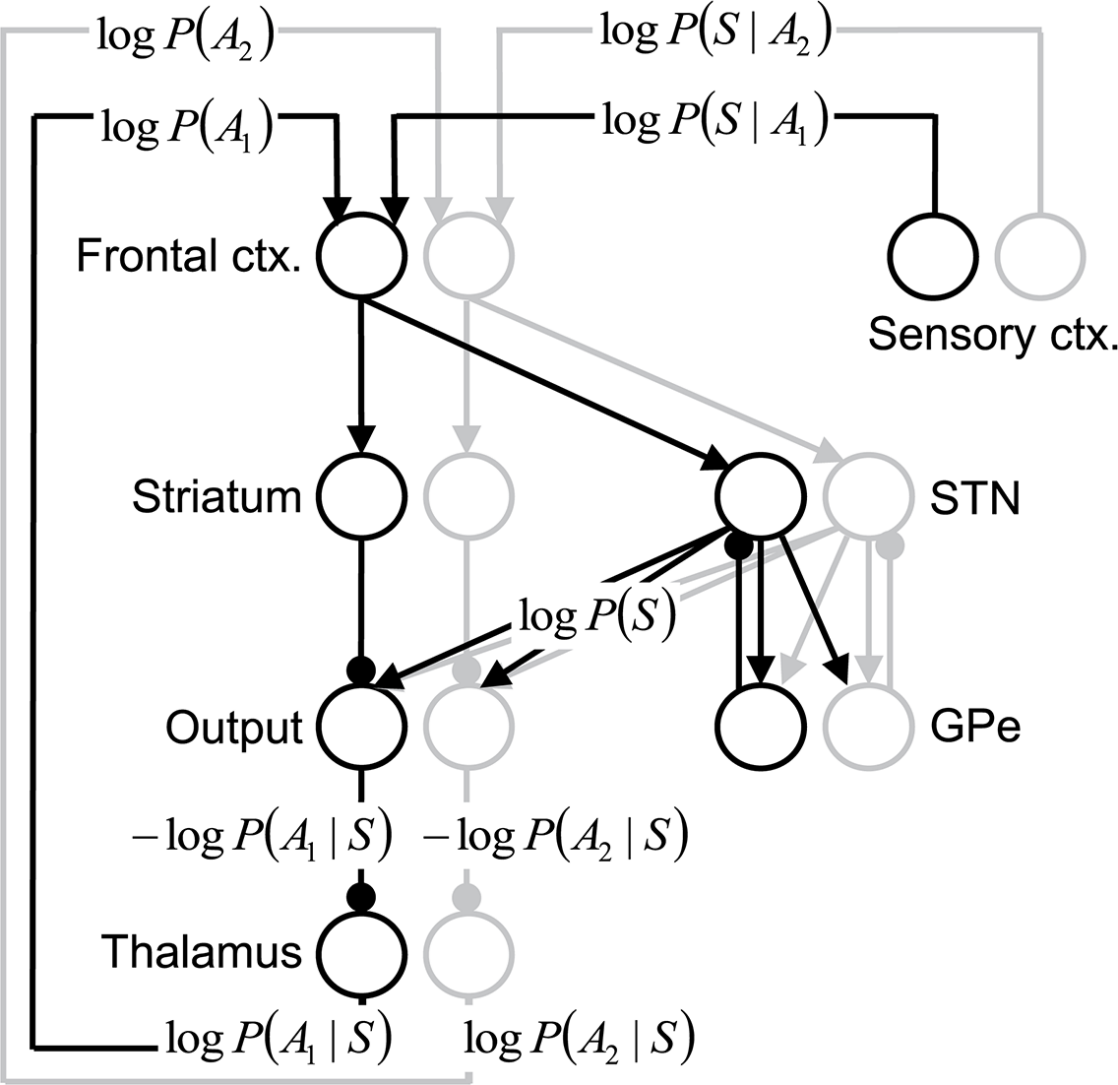
Mapping of Bayes’ theorem onto a subset of the cortico-basal-ganglia-thalamic circuits. Black and grey circles denote neural populations selective for two sample actions (*A*_*1*_ or *A*_*2*_), and the labels next to the circles indicate their anatomical correlates. Arrows denote excitatory (glutamatergic) connections, while lines ended with circles denote inhibitory (GABAergic) connections. The following abbreviations have been used: ctx.–cortex, STN–subthalamic nucleus, GPe–external globus pallidus, Output–output nuclei of the basal ganglia, i.e. internal globus pallidus and substantia nigra pars reticulata.

Fig 1 illustrates how Eq 3 could be mapped onto a subset of cortico-basal-ganglia-thalamic circuits [10]. The model assumes that within the circuit there exist populations of neurons selective for different actions (shown in different colours in Fig 1). The notion that different actions could be subserved by discrete neuronal populations within cortico-basal-ganglia-thalamic circuits is supported by anatomical data demonstrating that these circuits are composed of partially segregated (and topographically organized) ‘loops’ [23]. It has been demonstrated that, for certain assumptions, log *P*(*S*|*A*_*k*_) is proportional to the activity of the sensory neurons selective for stimuli associated with action *A*_*k*_ being correct [9,24–26], so the term log *P*(*S*|*A*_*k*_) could be encoded directly in the activity of cortical sensory neurons. In the model framework in Fig 1, the neurons in the frontal cortex add the input from sensory neurons to the logarithm of the prior probability, which is provided by a feedback from the thalamus, thus they perform the addition in Eq 3.

The logarithm of the normalization term is computed in the model in a circuit of reciprocally connected STN and GPe neurons, and this computation is described in detail in the next subsection. The output nuclei of the basal ganglia receive excitation from STN (which, in the model, is proportional to log *P*(*S*)) and inhibition from the cortex via the striatum (which, in the model, is proportional to log *P*(*A*_*k*_) + log *P*(*S*|*A*_*k*_)), and subtract these two inputs; thus, according to Eq 3, their activity is proportional to –log *P*(*A*_*k*_|*S*). The output nuclei send inhibitory signals to the thalamus, so the activity in the thalamus is proportional to the logarithm of the posterior probability, i.e. log *P*(*A*_*k*_|*S*). Finally, the logarithm of the posterior probability is sent back from the thalamus to the frontal cortex as it becomes the basis of the computation (or prior log *P*(*A*_*k*_)) in the next time step.

The model described so far assumes that certain neural populations have activity proportional to the logarithms of probabilities, but these quantities are negative (as probabilities are smaller than one). This problem can be solved by assuming that the firing rates are proportional to the logarithms of probabilities increased by a constant *c* (we discuss the required value of this constant in the Results section). Eqs 5–9 below describe computations performed by each of the nuclei in the model:

$$SEN_{k}=\log P\left(S|A_{k}\right)+c$$ (5)

$$CTX_{k}=\{\begin{array}{l} \log\;P\left(A_{k}\right)+c+SEN_{k}\;\text{at the first interval}\\ TH_{k}\left(t-1\right)+SEN_{k}\;\text{at subsequent intervals} \end{array}$$ (6)

$$STN=\log\sum_{k=1}^{N}\exp CTX_{k}$$ (7)

$$OUT_{k}=–CTX_{k}+STN$$ (8)

$$TH_{k}=c–OUT_{k}$$ (9)

In the above equations *SEN*_*k*_, *CTX*_*k*_, *OUT*_*k*_ and *TH*_*k*_ respectively denote the firing rates of populations of sensory cortical neurons, frontal cortical neurons, basal ganglia output nuclei neurons, and thalamic neurons, selective for alternative *k*. At the start of the trial, the frontal cortical neurons are initialized to the logarithms of initial prior probabilities of actions, and subsequently they receive feedback equal to thalamic activity in the previous time step i.e. *TH*_*k*_(*t*–1). The *STN* term denotes the sum of activities across all STN neurons, while we denote the activity of STN the neurons selective for action *A*_*k*_ as *STN*_*k*_, i.e.

$$STN=\sum_{k=1}^{N}STN_{k}$$ (10)

Thus, according to Eq 8, each neural population in the output nuclei in the model receives input from all populations in the STN, in agreement with experimental data suggesting that the axonal projections of STN neurons are relatively diffuse [27].

We will describe in the next subsection how the activity described by Eq 7 could arise in the STN, but first let us show that the model described in Eqs 5–9 correctly updates probabilities. At the first time-step, the activity of frontal cortical neurons is equal to (according to Eqs 5 and 6):

$$CTX_{k}=\log P\left(A_{k}\right)+\log P\left(S|A_{k}\right)+2c$$ (11)

According to Eqs 7, 11 and 2, the activity in the STN is:

$$\begin{array}{l} STN=\log\sum_{k=1}^{N}\exp\left(\log P\left(A_{k}\right)+\log P\left(S|A_{k}\right)+2c\right)=\\ \;=\log\sum_{k=1}^{N}P\left(A_{k}\right)P\left(S|A_{k}\right)\exp\left(2c\right)=\\ \;=\log P\left(S\right)+2c \end{array}$$ (12)

Constants 2*c* then cancel while computing the activity in the output nuclei (using Eqs 8, 11, 12 and 3):

$$OUT_{k}=-\log P\left(A_{k}\right)-\log P\left(S|A_{k}\right)-2c+\log P\left(S\right)+2c=-\log P\left(A_{k}|S\right)$$ (13)

We have shown that at the end of the first time interval, the model computes the posterior probabilities of actions. Since the posterior probabilities are then fed back to frontal cortical neurons as a prior for the next interval, it can be shown using analogous calculations that the network correctly computes the posterior probability in every subsequent interval.

### Computation of the normalization term

We now describe the conditions under which the activity in STN is proportional to the logarithm of the normalization term. Bogacz and Gurney [9] have shown that the STN-GPe circuit with the architecture shown in Fig 1 would produce activity of STN that is given in Eq 7, if and when the neural populations in STN and GPe had the following relationships between their inputs and their firing rates:

$$STN_{k}=\exp\left(CTX_{k}-GP_{k}\right)$$ (14)

$$GP_{k}=STN-\log STN$$ (15)

In the above equations, *GP*_*k*_ denotes the firing rates of GPe neurons selective for action *A*_*k*_ (in the original model [9] the GPe neurons were assumed to belong to a single cell type). The STN neurons receive excitation from cortex and inhibition from GPe, so their total input is *CTX*_*k*_−*GP*_*k*_, thus Eq 14 implies that the STN neurons in the model have exponential input-output relationships (often termed ‘f-I curves’ in empirical studies). The GPe neurons receive input from STN, but this input is coming in Fig 1 from STN neurons selective for all actions which we denote by *STN* without a subscript (see Eq 10). Eq 15 implies that the GPe provides inhibition proportional to *STN*–log *STN*.

Before giving a mathematical proof for how the STN-GPe circuit computes Eq 7 in the model, let us first provide an intuition. Starting from the right end of Eq 7, the frontal cortical activity *CTX*_*k*_ is provided to the STN in the model by the cortico-subthalamic pathway (see Fig 1). The exponentiation is performed by the STN neurons (cf. Eq 14). The summation is achieved due to the diffuse projections from the STN: in the model each neural population in the output nuclei receives input from all populations in the STN, hence the neurons in the output nuclei can sum the activity of STN populations. The only non-intuitive element of the computation of Eq 7 is the logarithm–this comes from the interactions between STN and GPe, as shown below.

We now present a sequence of simple mathematical operations that show that Eqs 14 and 15 imply Eq 7. Substituting Eq 15 into 14 gives:

$$STN_{k}=\exp\left(CTX_{k}-STN+\log STN\right)$$ (16)

Using the property of exponentiation *e*^*a+b*^ = *e*^*a*^*e*^*b*^ we obtain:

$$STN_{k}=\exp\left(CTX_{k}\right)\exp\left(-STN\right)STN$$ (17)

Summing over *k* and using the definition of *STN* (given in Eq 10) we obtain:

$$STN=\sum_{k=1}^{N}\exp\left(CTX_{k}\right)\exp\left(-STN\right)STN$$ (18)

Taking the logarithm of Eq 18 we get:

$$\log STN=\log\sum_{k=1}^{N}\exp CTX_{k}-STN+\log STN$$ (19)

log *STN* cancel on both sides in Eq 19, and moving *STN* to the left side we see that the sum of activities of all STN neural populations is equal to the required value of Eq 7:

$$STN=\log\sum_{k=1}^{N}\exp CTX_{k}$$ (20)

Eqs 14 and 15 thus describe the predictions of this model on the response properties of STN and GPe neurons, respectively. Bogacz and Gurney [9] have shown that the published f-I curves of STN neurons [28,29] indeed follow the exponential function precisely up to the firing rate of 135 spikes per second (STN neurons are unlikely to fire at higher rates *in vivo*). In the next Section, we investigate whether the properties of GPe neurons match those required to compute Eq 15.

## Results

We start by analysing in detail the computations in GPe required by the optimal action selection model, and by giving further insight into our initial hypotheses on how this computation could be performed [9]. We then show how the key aspects of our hypothesis match experimental data. Using simulations of an optimised computer model embedding realistic f-I curves of neurons for each sub-population, we demonstrate that GPe can perform computations required for optimal action selection. We finally report the dynamical properties of such network for varying cortical inputs, and discuss how the connections between GPe and striatum can be incorporated into the model.

### Computations in GPe required for optimal action selection

**Fig 2:**
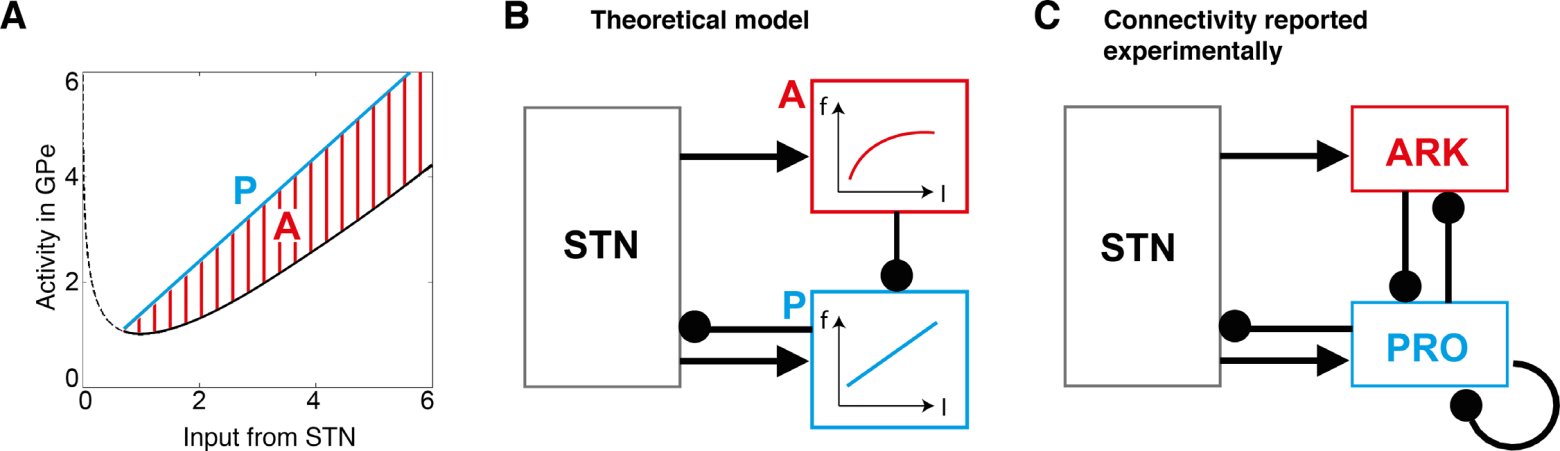
Computation of function *STN*–log *STN* in a microcircuit containing two distinct populations of GPe neurons. **(A)** Decomposition of function *STN*–log *STN* (shown in black) into a difference between a Prototypic function (P, shown in blue) and Adjustment (A, corresponding to red striped area). **(B)** A hypothetical neural circuit computing *STN*–log *STN*. Rectangles denote neural populations, arrows denote excitatory connections, while lines ended with circles denote inhibitory connections. P denotes the neural population with f-I curve corresponding to function P from the left panel. Activity of neurons P is adjusted by inhibition from population denoted by A with f-I curve corresponding to the red area from the left panel. **(C)** Experimentally-derived connectivity between major cell types in STN-GPe circuit. PRO, prototypic GPe neurons; ARK, arkypallidal neurons.

The model described in the previous section predicts that the GPe neurons send an inhibitory signal to the STN that is proportional to *STN*–log *STN*. The black curve in Fig 2A illustrates the shape of this function. It is worth clarifying that the axes in Fig 2A are expressed in the units of log of probability [25], which are related to the units of firing rate and input current through scaling factors (discussed later) because the model assumes that the firing rates are proportional to the probabilistic quantities they represent. As shown in Fig 2A, the function *STN*–log *STN* diverges to infinity for very low *STN* input. However, such very low values of *STN* input are not biologically relevant because STN neurons are autonomously active [30,31]; GPe will thus receive input from STN even when STN itself does not receive any organised excitatory input. The lower bound on *STN* is provided by Eq 20 –please note that *CTX*_*k*_ cannot be negative thus *STN* ≥ log *N*. The lowest possible number of choice alternatives is 2, thus *STN* ≥ log 2 ≈ 0.7.

The upper bound on *STN* is provided by Eq 12; please note that log *P*(*S*) cannot be positive thus *STN* ≤ 2*c*. Because the upper bound depends on constant *c* we now consider its value. Recall that constant *c* was added in the model to the activity of the neurons representing logarithms of probabilities to ensure their firing rates are not negative. Nevertheless for any value of *c*, for sufficiently low probability *p*, the value of log *p* + *c* will be negative, so such low probabilities will not be represented in the model. Thus the value of constant *c* determines the lowest probability of action that can be represented in the model. It has been demonstrated that humans can represent prior probabilities of actions as low as 0.05 [32]. If we wish the model to represent the probability of 0.05, then *c* needs to be around 3, as log 0.05 + 3 ≈ 0. If we set *c* to 3, then the upper bound on STN considered above becomes *STN* ≤ 2*c* = 6. In summary, we will consider the relevant range of *STN* for which GPe needs to compute *STN*–log *STN* to be from around 0.7 to 6. The black curve in Fig 2A is solid in this range, while it is dashed outside it.

In the relevant range *STN* ϵ (0.7, 6), function *STN*–log *STN* is non-monotonic, i.e. it initially decreases (for *STN* < 1) and then increases (for *STN* > 1), so it is very unlikely that neurons with such an f-I curve would exist, and thus that this function could be computed by single neurons. Even ignoring the non-monotonicity, which occurs on only a small part of the relevant range, the function *STN*–log *STN* is convex on its whole range, i.e. the larger the input, the larger is its rate of growth. By contrast, the previously published f-I curves of GPe neurons were only convex in a very narrow range of small input currents, while on the majority of their range they were linear or concave, i.e. they were decreasing their rate of growth for larger inputs [33–35]. Hence, the published data suggest that it is unlikely that individual GPe neurons, or a single type of GPe neuron, could compute even the monotonic part of function *STN*–log *STN*. Theoretically, however, this function could be computed in a microcircuit composed of two populations of GPe neurons with distinct activities and connections. Fig 2A shows how the function *STN*–log *STN* could be represented by a difference of two functions: a 'Prototype' (P) function, shown by a blue line, and an 'Adjustment' (A) function, shown by a red striped area. Therefore, the function *STN*–log *STN* could be computed by two neural populations, ‘P’ and ‘A’, with the connectivity shown in Fig 2B, and with f-I curves corresponding, respectively, to the blue line and the height of red striped area (i.e. the difference between blue and black lines) in Fig 2A (such f-I curves are monotonic and non-convex). In this architecture, both populations P and A receive input from STN. Population A transforms this input via its non-linear f-I curve and sends inhibition to neurons P thus adjusting their response. In this architecture only the neurons in population P have activity proportional to *STN*–log *STN*, so *only* they project back to STN.

### Comparison of connectivity of GPe neurons with model predictions

GABAergic projection neurons in the GPe can be divided into two major cell types, termed prototypic and arkypallidal, on the basis of their distinct firing *in vivo*, molecular profiles and structure [18–20]. These sub-populations exhibit clearly distinct connectivity within the STN-GPe network (Fig 2C), as derived from empirical studies in rodents [19,20] and previous computational modelling of empirical data [36]. Interestingly, this pattern of connectivity resembles that of the model computing function *STN*–log *STN* (cf. Fig 2B). In particular, only one of the GPe populations, i.e. the prototypic neurons (but not arkypallidal neurons) send projections back to STN [19,20]. Additionally, recent computational modelling of effective connectivity in the STN-GPe network suggest that both prototypic and arkypallidal neurons receive input from STN [36]. Thus, in summary, the observed pattern of GPe connectivity (Fig 2C) includes all the connections present in the model computing function *STN*–log *STN* (Fig 2B), but it also includes two additional connections (i.e. that between prototypic neurons and that from prototypic to arkypallidal neurons) and we will address their roles below.

### Comparison of response properties of GPe neurons with model predictions

We investigated whether the f-I curves of GPe neurons have characteristics required for computation of function *STN*–log *STN*. The first characteristic we expected was that f-I curves of GPe neurons should have linear or logarithmic shape, allowing them to jointly compute the function *STN*—log *STN*. Then we sought to explore if the populations 'P' and 'A' introduced in our theoretical model could correspond to the prototypic and arkypallidal neurons reported experimentally. Such a correspondence would require further two characteristics in their f-I curves. For low levels of input from STN, the average firing rate of prototypic neurons should be higher than that of arkypallidal neurons. This requirement arises because the “adjustment” to the firing rate of the 'P' sub-population (red area in Fig 2A) is only necessary for high inputs, so one could expect arkypallidal neurons to have a firing rate closer to zero for low *STN*. Furthermore, one would expect that the responses of prototypic neurons to be more linear than those of arkypallidal neurons (as the two populations in Fig 2B compute the linear and logarithmic functions in Fig 2A).

**Fig 3:**
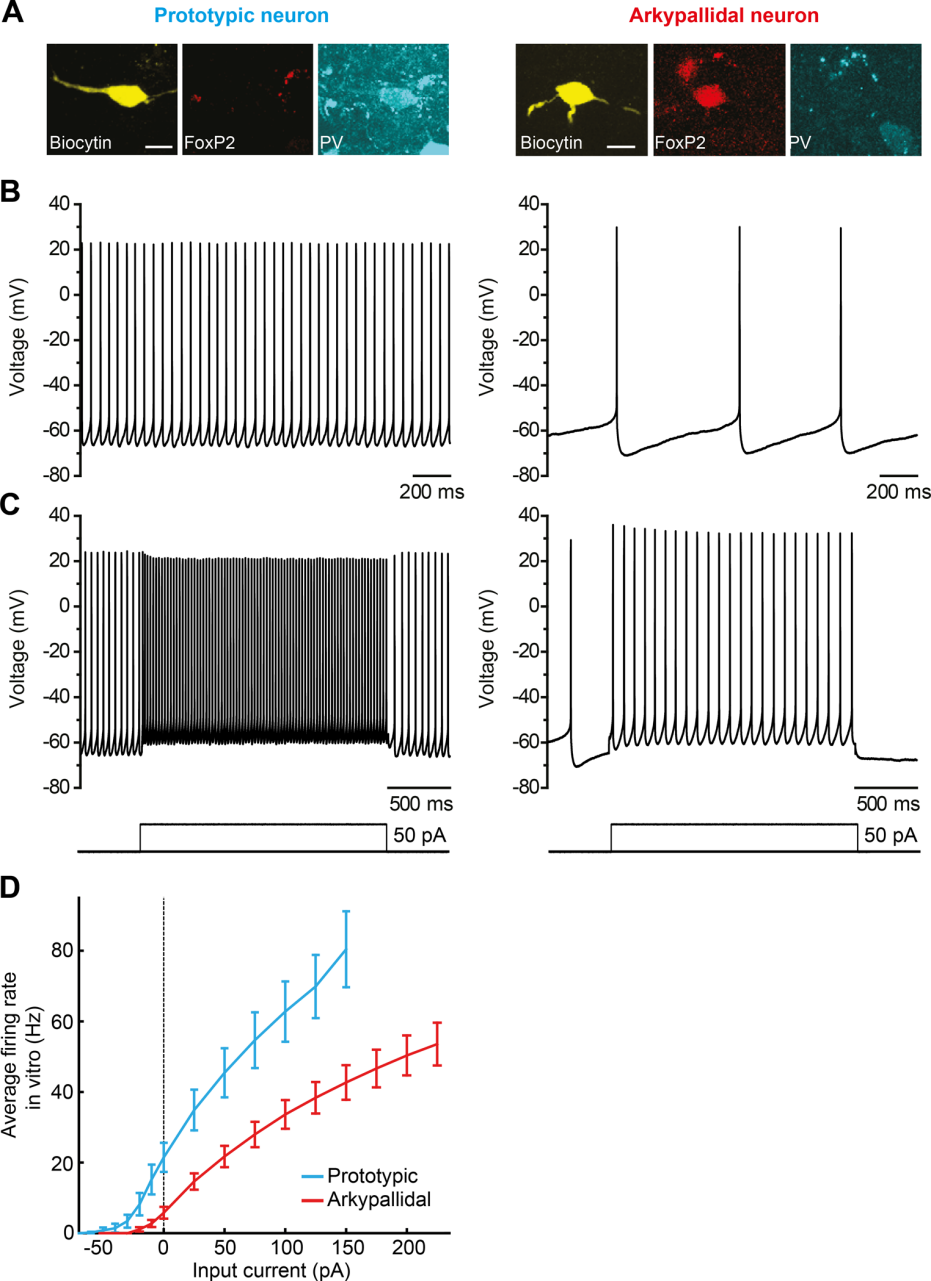
Autonomous firing and driven responses of molecularly-identified GPe neurons. **(A)** After recording, all neurons were labelled with biocytin, recovered and tested for expression of parvalbumin (PV) and forkhead box protein 2 (FoxP2). Prototypic GPe neurons (left) expressed PV but not FoxP2, whereas arkypallidal neurons (right) expressed FoxP2 but not PV. Scale bars = 20 μm. **(B)** Typical examples of the autonomous firing of a prototypic neuron (left) and an arkypallidal neuron (right). **(C)** Typical examples of the driven activity of a prototypic neuron (left) and arkypallidal neuron (right). Same neurons as shown in A and B. **(D)** Average f-I curves of prototypic and arkypallidal neurons recorded *in vitro*. The error bars show the standard error of the mean.

The f-I curves of molecularly-identified prototypic and arkypallidal neurons have been recently measured experimentally by Abdi et al. [19] using perforated patch-clamp recordings in rat brain slices. Experimental procedures in that study were conducted either in Oxford in accordance with the Animals (Scientific Procedures) Act, 1986 (United Kingdom), or in Bordeaux according to institutional guidelines and the European Communities Council Directive 86/609/EEC and its successor 2010/63/EU. During these recordings, the slices were perfused continuously with oxygenated artificial cerebrospinal fluid at 35°C–37°C. Firing rates were recorded as a function of injected current for 18 prototypic neurons (expressed PV but not FoxP2) and 18 arkypallidal neurons (expressed FoxP2 but not PV) (Fig 3A). In these measurements, the depolarising current injection was gradually increased in magnitude until a point at which the neuron was unable to follow (by firing well-defined action potentials). To avoid excluding any neurons from the analysis, the average f-I curve for a given type of neuron was computed for the range in which all the studied neurons of this type were able to respond. All prototypic and arkypallidal neurons responded to currents up to 150 pA and 225 pA respectively, and hence the average f-I curves were computed up to these values. For each value of current, the firing rate was measured over 2 s interval during which the current was injected (Fig 3C). Additionally, the rate of autonomous firing was measured (Fig 3B), that is, the firing present with 0 current injection in the presence of receptor antagonists [19].

**Fig 4:**
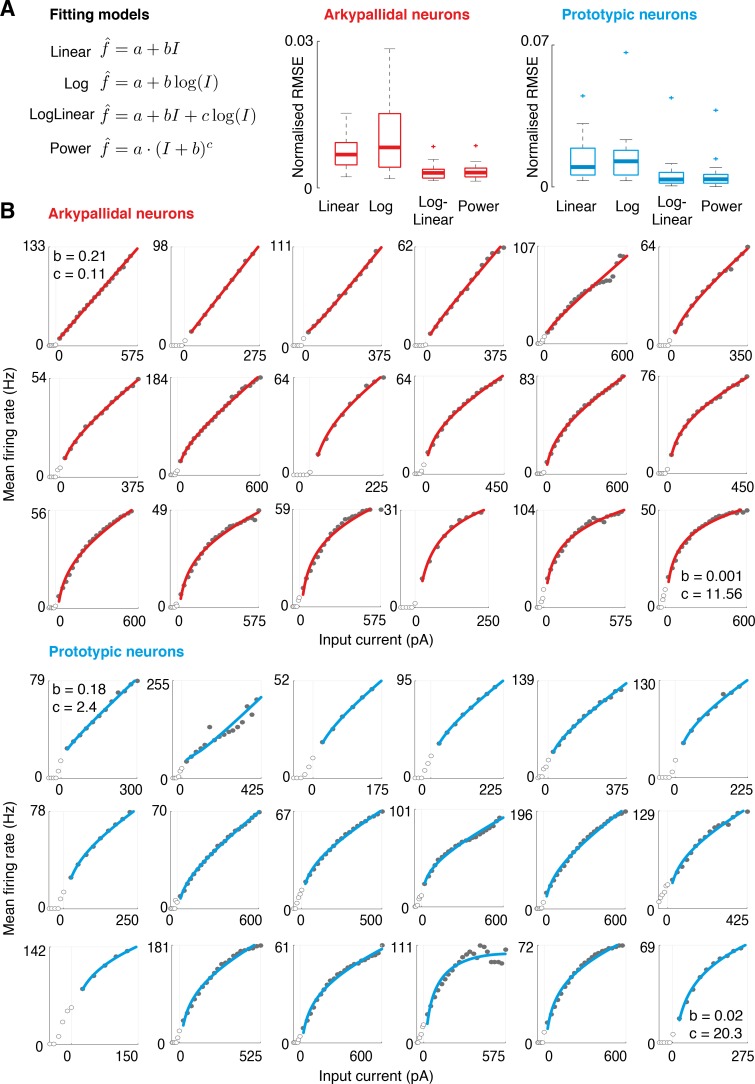
Linearity and non-linearity of f-I curves of GPe neurons. **(A)** Comparison of quality of fits of f-I curves of arkypallidal or prototypic neurons to 4 different functions (linear, logarithmic, a combination of logarithmic and linear, or a power function). The fits were assessed by root mean squared error (RMSE). Note that the best fits (lowest RMSE values) arose from using the combination of logarithmic and linear as well as from the power function. Plots indicate medians plus IQRs for all neurons of each type. **(B)** Individual fits for all f-I curves recorded in arkypallidal (n = 18) and prototypic (n = 18) neurons. Traces are sorted from most linear (top left) to logarithmic (bottom right) for each population. Only positive responses are considered, since log *x* is undefined for *x≤0*. In the displays showing the most linear and the most logarithmic neurons, the values of coefficients in fitted function *f* = *a* + *bI* + *c*log*I* are printed. The logarithmic coefficient *c* has relatively high values even for the most linear neurons, because function log*I* has much smaller range for a given input than the linear function *I*.

The average f-I curves of all prototypic and all arkypallidal neurons are shown in Fig 3D, and the f-I curves of individual neurons are shown in Fig 4B (the data is provided in S1 Table). One characteristic we expected was that, for low values of excitatory input (nominally from STN) the activity of prototypic neurons should be higher than that of the arkypallidal neurons. This expectation agrees with the experimental data (see Fig 3D), as the average rate of the autonomous firing of prototypic neurons (18.0 spikes/s) was higher than that of arkypallidal neurons (5.1 spikes/s), and this difference was highly significant (p = 0.0001, unpaired t-test).

To test the linear or logarithmic behaviour of GPe neurons, we compared the fitting quality of different functions to the f-I curves of each neuron. We obtained the best fitting parameters *a*, *b* and *c* for different functions (linear *f* = *a + bI*, logarithmic *f* = *a + b*log*I*, a combination of both functions *f* = *a* + *bI + c*log*I*, or a power function *f* = *a*(*I* + *b*)^*c*^) by minimizing the root mean squared error (RMSE) between the actual and the predicted firing rates over the range of positive input currents for each neuron. To help visually asses the shape of f-I curves in Fig 4B, we ordered the neurons according to a bias for linear fit, defined as a ratio of RMSE between linear and logarithmic fits. We found that all f-I curves can be well explained by a combination of linear and logarithmic functions (shown as solid curves in Fig 4B; average r^2^ for arkypallidal and prototypic neurons were 0.998 and 0.994 with standard deviations 0.0041 and 0.013). Furthermore, the f-I curves ranged from almost fully linear to clearly logarithmic (i.e. very low values of linear coefficient *b* in function *f* = *a* + *bI + c*log*I*). The power function could describe the f-I curves equally well (Fig 4A) as both a power function and a combination of linear and logarithmic functions can take a similar, concave shape. To verify whether the diversity of the shapes of f-I curves is not an artefact stemming from differences in the recording quality of individual neurons, we computed the correlation between the bias for linear fit and the series resistances during perforated-patch recordings, which was not significant.

Contrary to our expectations, we did not find statistically significant differences in the linearity of the f-I curves of prototypic and arkypallidal neurons (the linearity was quantified as the ratio of RMSE between linear and logarithmic fits). Indeed, both populations included neurons that lied within a continuum ranging from highly linear to highly logarithmic (Fig 4B). Even though this third expected characteristic was not present, it is striking that the f-I curves of the GPe neurons range from the linear to the logarithmic, which are the two components of Eq 15 that describe the predicted computation in GPe.

### Connections between prototypic GPe neurons linearize their response profile

**Fig 5:**
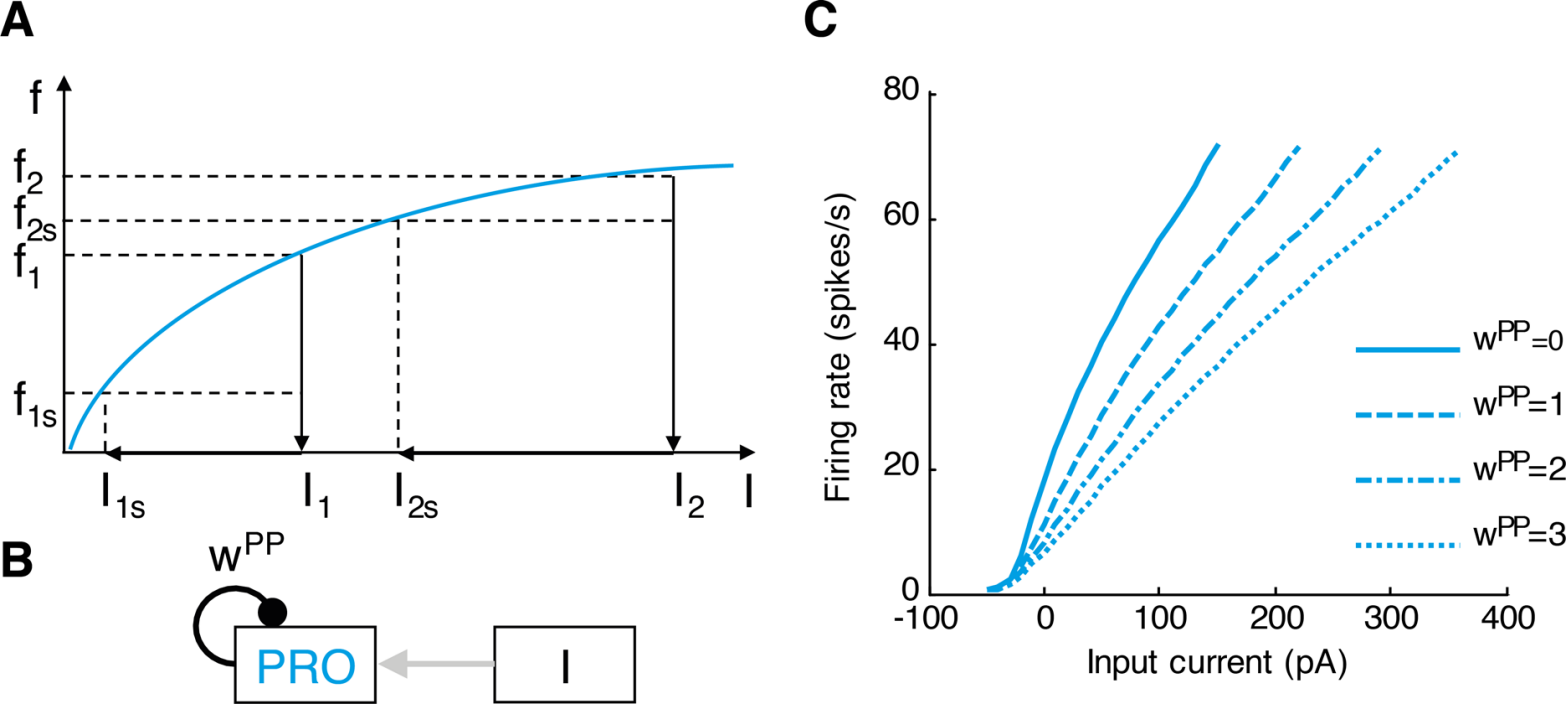
The connections between prototypic neurons linearize their response profiles. **(A)** A schematic diagram illustrating how mutual inhibition linearizes the response profile (see main text). **(B)** A model of a population of prototypic neurons, PRO, receiving excitatory input (I) and mutual inhibition (average weight *w*^*PP*^). **(C)** The resulting response profiles for different levels of inhibition within the population (when *w*^*PP*^ = 0, there is no mutual inhibition). Note the linearization of the population response with increasing mutual inhibition.

Although not all prototypic neurons had linear response curves, their reciprocal connections may contribute to adjusting the shape of their response profiles (these connections have been experimentally observed, but were not initially included in our theoretical network shown in Fig 2B). We now show that the mutual inhibitory connections within the population of prototypic neurons linearize their response profile. The intuition for this effect is provided in Fig 5A, which shows a hypothetical concave (or saturating) f-I curve that qualitatively resembles the average empirical response of prototypic neurons to depolarizing current injections (Fig 3D). Let us consider two cases of excitatory input currents. A small input current *I*_1_ without the mutual inhibition would produce firing *f*_1_. However, with mutual inhibition, the overall input is reduced proportionally to *f*_1_ to a smaller value *I*_1*s*_, which gives a lower firing *f*_1*s*_. Analogously, for a higher input current *I*_2_ the mutual inhibition reduces the firing from *f*_2_ to *f*_2*s*_. Please note that the reduction in firing due to mutual inhibition in the case of small input, i.e. *f*_1_ –*f*_1*s*_, is more pronounced than for the case of large input, i.e. *f*_2_–*f*_2*s*_. Since the reduction is more pronounced in the less linear part of the curve than the more linear one, the mutual inhibition linearizes the response profile.

To quantify the effect of mutual inhibition on response properties of prototypic neurons, we modelled the responses of a population of prototypic neurons receiving an external excitatory input *I* and inhibiting the neurons within the population (Fig 5B). Throughout the paper, we denote the strength of connections between population *X* and *Y* by *w*^*XY*^, where *X* and *Y* can be *P* for prototypic, *A* for arkypallidal or *S* for STN. Thus, the dynamics of this population of prototypic neurons with mutual inhibition are described by the following equation:

$$P\dot{R}O=f_{P}\left(I-w^{PP}PRO\right)-PRO$$ (21)

In the above equation, function *f*_*P*_(*x*) is based on the average f-I curve of prototypic neurons (see Fig 3D), and was defined in the following way. If *x* was equal to a current used in the in vitro experiment for all prototypic neurons, *f*_*P*_(*x*) was simply equal to the average firing rate for this current. If *x* was between two currents tested in experiment, *f*_*P*_(*x*) was found using linear interpolation. If *x* was below the lowest current tested in experiment, *f*_*P*_(*x*) was set to 0. Finally, if *x* was above the range on which the average f-I curves were computed (see above), *f*_*P*_(*x*) was set to the firing at the maximum current used with all prototypic neurons, but in all analyses below, we ensure that we do not present results relying on this value, as in this case the f-I curve is undetermined.

The last term (–*PRO*) in Eq 21 is a decay term. The value to which the variable *PRO* converges can be found by setting the left hand side of Eq 21 to 0, because at convergence, the value of *PRO* does not change. Thus we find that at convergence *PRO* = *f*_*P*_(*I* − *w*^*PP*^ *PRO*). So in this model, when *w*^*PP*^ = 0, the activity *PRO* is equal to the activity determined by the f-I curve for given input *I*, while when we increase *w*^*PP*^, we can investigate the effect of mutual inhibition on the response profile.

The activity of the prototypic neurons at convergence as a function of external input is shown in Fig 5C for different values of mutual inhibition. As the strength of the connection between prototypic neurons (*w*^*PP*^) increases, their response becomes more linear. To quantify it, we computed squared correlation (*R*^2^) between *I* and *PRO* for inputs *I*>0. The *R*^2^ was increasing with *w*^*PP*^, namely it was equal to 0.988, 0.994, 0.996 and 0.997 for *w*^*PP*^ equal to 0, 1, 2 and 3 respectively.

### Computations in a network of arkypallidal and prototypic GPe neurons

**Fig 6:**
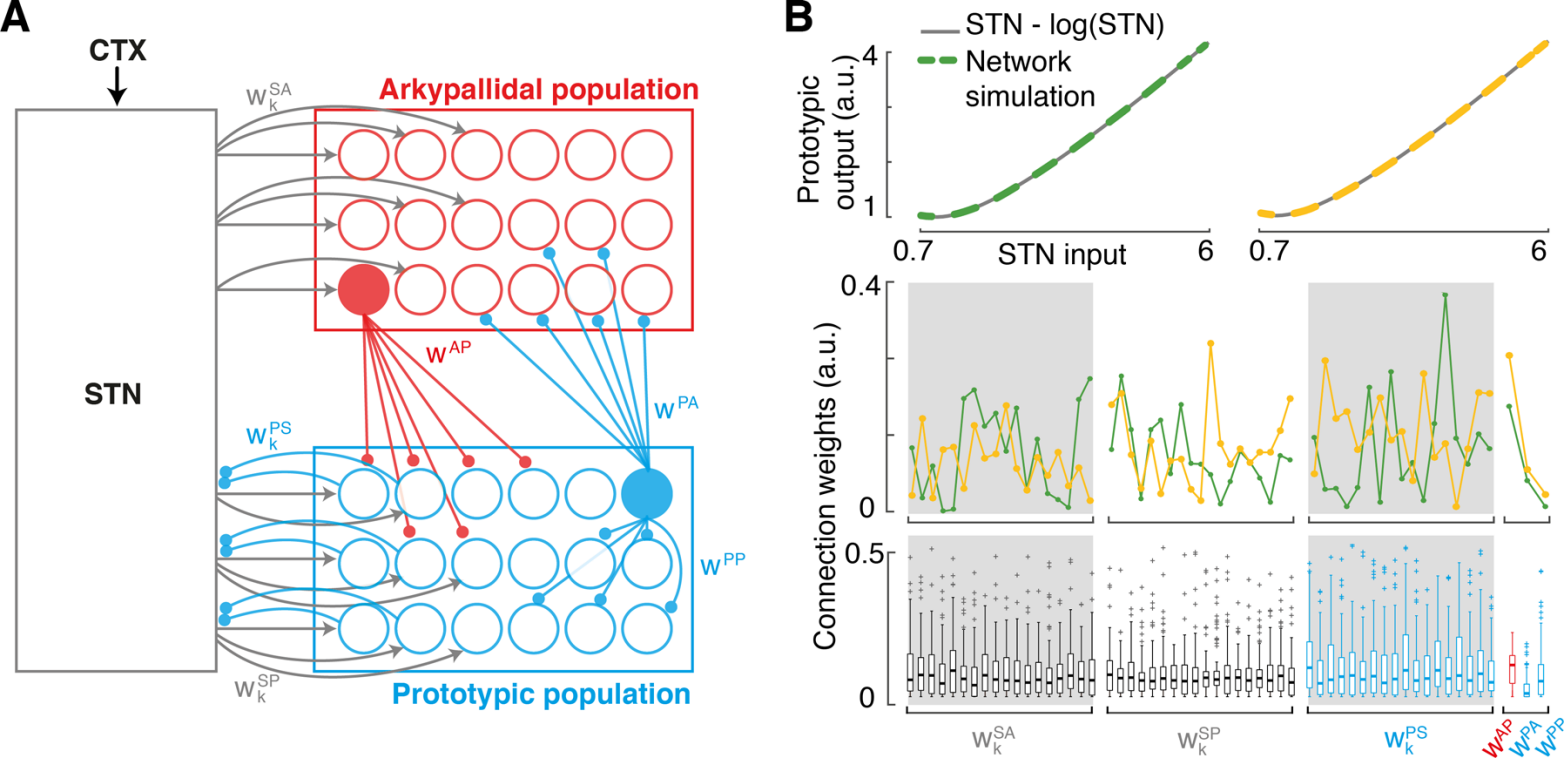
Computational model of STN-GPe network. **(A)** Architecture of the model including all 18 prototypic and arkypallidal neurons recorded experimentally, and connections within GPe and with STN. *w*_*i*_^*XY*^ denotes the strength of connections between population *X* and *Y*. Sub-indices indicate the individual neuron associated with each weight. Inner GPe weights linking arkypallidal and prototypic neurons are assumed identical for all connections, hence represented by a single value. **(B)** Illustrative examples of inhibition sent from GPe to STN i.e. $\sum_{i=1}^{18}w_{i}^{PS}PRO_{i}\left(t\right)/\alpha_{S}$for different optimal weights (dashed yellow and green), and comparison with the desired function *STN–*log *STN* throughout the input range considered ([0.7–6]). Middle plot shows two sets of weight parameters corresponding to the top display, and the bottom display shows the range of weight parameters found in 100 runs. Weights for each population are organised from the most linear neuron’s to the most logarithmic neuron’s (left to right), highlighting the lack of preferential connections to linear or logarithmic neurons.

To test our main prediction, namely that a network of GPe neurons is capable of sending inhibition to STN to instantiate the function *STN*–log *STN*, we next investigated the behaviour of a realistic, multi-dimensional network including all the different neurons recorded in the arkypallidal and prototypic populations. We built a computational network embedding the 18 arkypallidal and 18 prototypic neurons with f-I curves based on experimental data, and all corresponding connections (Fig 6A). This included 36 excitatory connections from STN to each neuron in these two GPe sub-populations (*w*_*i*_^*SA*^ and *w*_*i*_^*SP*^), and 18 inhibitory connections from each prototypic neuron back to STN (*w*_*i*_^*PS*^; these connections influence the cost function that will be introduced later). The model also included 18^2^ inhibitory connections from arkypallidal to prototypic neurons, 18^2^ inhibitory connections from prototypic to arkypallidal neurons, and 18^2^ inhibitory connections between prototypic neurons, but to reduce the high dimensionality of this system, we assumed similar levels of connectivity, and constrained the weights in each group to a single value (*w*^*AP*^, *w*^*PA*^ and *w*^*PP*^). Thus the dynamics of the simulated neurons was described by:

$$A\dot{R}K_{i}=f_{Ai}\left(18\cdot w_{i}^{SA}\alpha_{S}STN-\sum_{k=1}^{18}w_{}^{PA}PRO_{k}\right)-ARK_{i}$$ (22)

$$P\dot{R}O_{i}=f_{Pi}\left(18\cdot w_{i}^{SP}\alpha_{S}STN-\sum_{k=1}^{18}w_{}^{AP}ARK_{k}-\sum_{j=1}^{18}w^{PP}PRO_{j}\right)-PRO_{i}$$ (23)

In the above equations *f*_*Ai*_ and *f*_*Pi*_ denote the f-I curves based on experimental data for individual neurons (computed analogously to *f*_*P*_ described above). In addition, to prevent bias during the optimisation process, the STN input sent to GPe was assumed to stem from 18 identical neurons, which ensured a homogeneous number of neurons in all three populations, and normalised the weights between them to comparable ranges. For a given value of *STN*, these equations were solved until convergence.

In Eqs 22 and 23 the activity of STN is scaled by a constant *α*_*S*_ to translate it to appropriate units. As mentioned before, the variables of the model are expressed in the units of the logarithm of probability that are proportional to firing rates, and we denote the proportionality constant for STN by *α*_*S*_. Thus, the firing rate of STN neurons in [spikes/s], is *α*_*S*_*STN*. The value of *α*_*S*_ can be estimated on the basis of published f-I curves of STN neurons. In particular, for zero input, *STN* = exp(0) = 1, so *α*_*S*_ is equal to the autonomous firing rate of STN neurons. Hence we set *α*_*S*_ = 17 on the basis of average autonomous firing rate of 7 STN neurons for which the f-I curves have been published [28,29] and have been fitted with exponential functions [9].

We found the weights for the 57 connection parameter through constrained nonlinear optimisation, which minimised the difference between the overall inhibition sent back to STN from GPe and the desired function *STN*–log *STN* in the input range considered [0.7–6] (a.u.).

$$Cost=\sum_{STN=0.7}^{6}{\left(STN-\log\;STN-\sum_{i=1}^{18}w_{i}^{PS}PRO_{i}/\alpha_{S}\right)}^{2}$$ (24)

Similarly as above, the feedback from prototypic neurons is divided by *α*_*S*_ in Eq 24 to bring it back from [spikes/s] to the units of the model. We note that the precise value of *α*_*S*_ does not affect the ability to find weights of connections, because it just scales other free parameters (*w*_*i*_^*SA*^, *w*_*i*_^*SP*^, *w*_*i*_^*PS*^ in Eqs 22, 23 and 24, respectively), so changing *α*_*S*_ would only result in rescaling these parameters. We only include *α*_*S*_ to give the weight parameters the same scale, so their values could be compared.

We completed 100 runs of this optimisation process for weights initialised at random values ranging between 0 and 0.3. For all runs, the optimiser converged to set of weights that exhibited a behaviour following our desired output function (average *Cost* was 0.0214 with standard deviation 0.017). For each run, however, the parameters found showed clear differences, indicating that distinct set of weights are capable of producing similarly optimal output functions (i.e. close approximation of *STN*–log *STN*) in such a high dimensional system. Two examples of synaptic weights and resulting outputs from the network are shown in Fig 6B.

Regardless of differences between individual runs, we sought to further characterise whether any structure underlies the weights found through global optimisation. For example, one could expect the connections from STN to arkypallidal neurons to be stronger for neurons with logarithmic f-I curves, in accordance with our initial suggestion on how the function *STN*–log *STN* is computed (Fig 2B). The means and standard deviations of parameters found in all 100 optimisation runs is shown in Fig 6B (bottom panel), where the parameters in each group are sorted by how linear the f-I curve of each neuron is. Thus the preferential STN projections to logarithmic arkypallidal neurons would manifest in the figure in higher weights on the right of the first grey area. Fig 6B did not highlight any specific patterns, neither for linear nor for logarithmic neurons in any of the two populations of GPe neurons. This indicates that the network considered is so flexible that it contributes to optimal action selection without requiring clear patterns in the way linear and logarithmic neurons are interconnected.

Among the estimated weights, the lowest value was on average assigned to a connection from prototypic to arkypallidal neurons (Fig 6B). The low weight value was obtained probably because this connection was not necessary to compute function *STN*–log *STN* (it was not included in the hypothetical network of Fig 2B).

### Dynamics of STN-GPe network

Finally, we sought to evaluate the dynamics of the entire STN-GPe network in response to varying cortical input. We evaluated the transient behaviour of the network (weights were set to one solution found during the optimization) when considering realistic time constants for firing rates (*τ*) and realistic transmission delays (Δ*t*), as used in a previous model of this circuit [36]. In particular, we used the following time constant *τ*_*S*_ = 10 ms [37,38], *τ*_*A*_ = *τ*_*P*_ = 15 ms [39] and transmission delays Δ*t*_*SA*_ = Δ*t*_*SP*_ = 2.8 ms, Δ*t*_*PS*_ = 1.3 ms and Δ*t*_*PA*_ = Δ*t*_*PP*_ = Δ*t*_*AP*_ = 1 ms [37,39]. The dynamics of the model are described by:

$$\tau_{S}S\dot{T}N=\exp\left(CTX-\sum_{i=1}^{18}w_{i}^{PS}PRO_{i}(t-\Delta t_{PS})/\alpha_{S}\right)-STN$$ (25)

$$\tau_{P}P\dot{R}O_{i}=f_{Pi}\left(18\cdot w_{i}^{SP}\alpha_{S}STN(t-\Delta t_{SP})-\sum_{k=1}^{18}w^{AP}ARK_{k}(t-\Delta t_{AP})-\sum_{j=1}^{18}w^{PP}PRO_{j}(t-\Delta t_{PP})\right)-PRO_{i}$$ (26)

$$\tau_{A}A\dot{R}K_{i}=f_{Ai}\left(18\cdot w_{i}^{SA}\alpha_{S}STN(t-\Delta t_{SA})-\sum_{j=1}^{18}w^{PA}PRO_{j}(t-\Delta t_{PA})\right)-ARK_{i}$$ (27)

**Fig 7:**
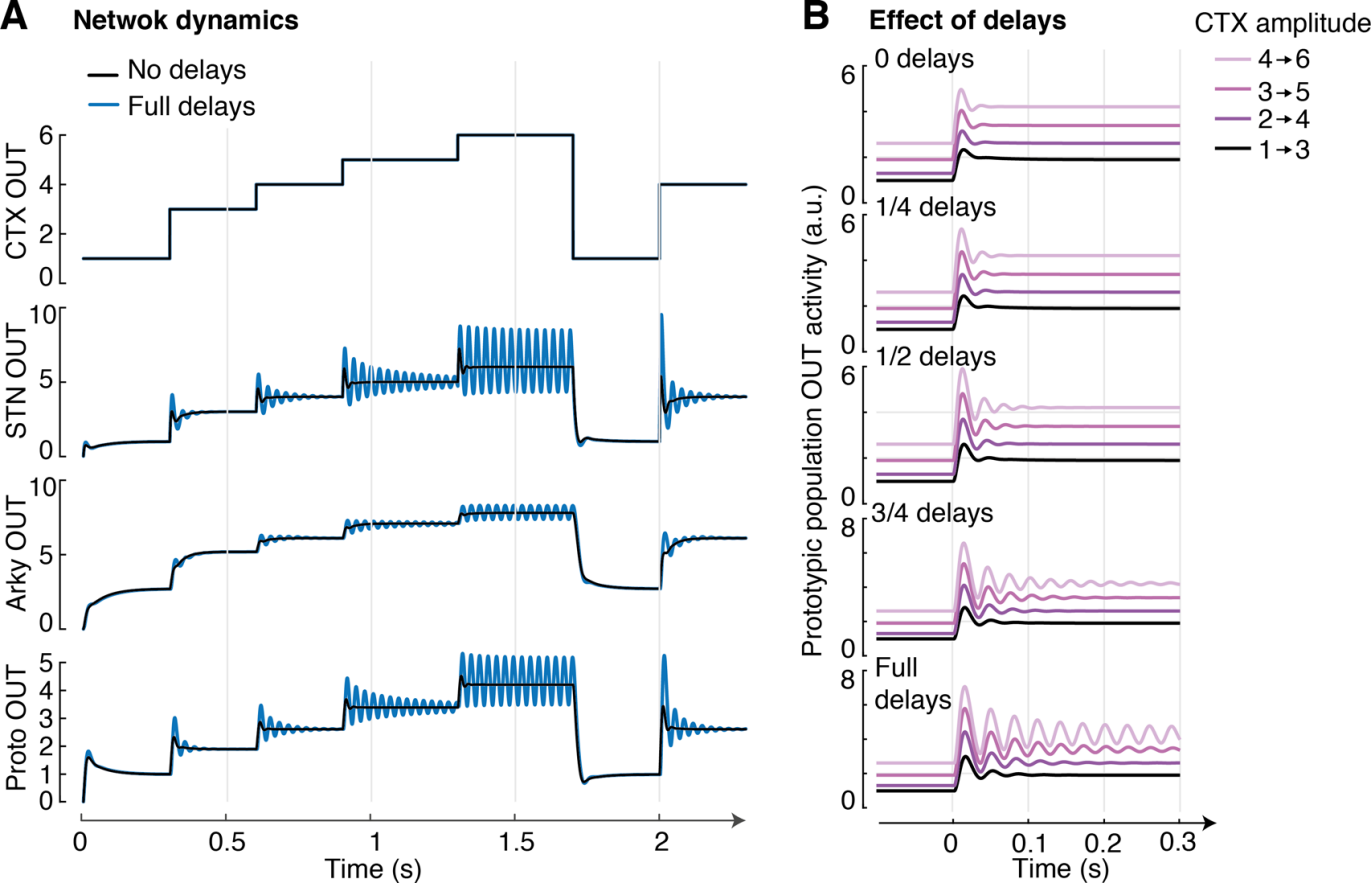
Dynamical behaviour of STN-GPe network. **(A)** Output responses of STN, Arkypallidal and Prototypic populations for a sequence of cortical (*CTX*) input steps ranging from 1 to 6 (a.u.). The output from arkypallidal neurons is taken as *w*^*AP*^<*ARK*_*i*_(*t*)>_*i*_, while the output from prototypic neurons was taken as $\sum_{i=1}^{18}w_{i}^{PS}PRO_{i}\left(t\right)/\alpha_{S}$. Black and blue traces correspond to the behaviour of the system without and with delays respectively. **(B)** Evaluation of the effect of delays in the system in response to varying cortical input. As delays increase (and delay values approach those reported in literature), the cortical input for which the system becomes unstable decreases.

We run multiple simulations and studied the behaviour of the system for varying cortical input values in the range *CTX* ϵ [0.7–6]. Since the above network includes only a single group of STN neurons (selective for a single action), the desired value to which STN should converge according to Eq 7 for *N* = 1 is simply *STN* = *CTX*. We found that the network properly converged to the expected value after a transient time period (the curves in the second display from the top in Fig 7A converge to the values in the top display), except for a very high cortical input (*CTX* = 6). For such high input the network started oscillating and did not converge. Oscillations are known to emerge in networks of mutually connected excitatory and inhibitory neural populations like the STN-GPe network under certain conditions [40,41]. One of necessary conditions is a strong reaction of neurons in one population to changes in activity of the other [40,41], and the oscillations emerged in Fig 7A for the high cortical input, as then the STN neurons were operating in steeper range of their exponential f-I curve, and thus were more reactive to their inputs from GPe.

In contrast, the same network always converged to the appropriate values in the absence of delays, as the delays are necessary for oscillations in the model of STN-GPe network [40,41]. We further analysed the influence of delays in this behaviour (Fig 7B). We compared the output of the network for delays values as reported in literature, without delays, and for different values in between, when cortical input values followed a step function (steps of 2 cortical units) starting at different input values (2, 3, 4 and 5). We found that the level of cortical input for which the system became unstable progressively decreased as delays increased.

### Analytic description of the computations in GPe

Beyond the simulated demonstration of how a GPe network, comprised of interconnected prototypic and arkypallidal neurons with experimentally observed f-I curves, is able to compute the function required for optimal action selection, it is also useful to show it analytically, as it will allow us to see in the next subsection how the connections between GPe and striatum can be incorporated into the model.

In order to demonstrate the computation analytically we consider a simplified model of the GPe circuit. We assume that the arkypallidal neurons have f-I curves described by a combination of linear and logarithmic terms (as Fig 4B shows that this function well describes experimentally observed curves):

$$f_{A}\left(I\right)=a_{A}+b_{A}I+c_{A}\log I$$ (28)

For simplicity, we will assume the response of prototypic neurons could be described as a linear function of their input, as we demonstrated that the mutual inhibitory connections among the prototypic neurons linearize their response profile (Fig 5).

$$f_{P}\left(I\right)=a_{P}+b_{P}I$$ (29)

We consider a network with the simplified connectivity shown in Fig 2B. Thus we do not consider connections among the prototypic neurons as their role has already been incorporated in assuming linear *f*_*P*_, and we do not consider connections from prototypic to arkypallidal neurons, as Fig 6B shows that this connection had a relatively low weight when the GPe network computed *STN*–log *STN*. The activity of prototypic neurons becomes:

$$\begin{array}{l} PRO=a_{P}+b_{P}\left(w^{SP}STN-w^{AP}ARK\right)=\\ \;a_{P}+b_{P}\left(w^{SP}STN-w^{AP}a_{A}-w^{AP}b_{A}w^{SA}STN-w^{AP}c_{A}\log w^{SA}STN\right) \end{array}$$ (30)

Rearranging the term we obtain:

$$PRO=\left(b_{P}w^{SP}-b_{P}b_{A}w^{AP}w^{SA}\right)STN-b_{P}c_{A}w^{AP}\log w^{SA}STN+a_{P}-b_{P}a_{A}w^{AP}$$ (31)

It is easy to see that the output of prototypic neurons *w*^*PS*^*PRO* will become equal to *STN*–log *STN*, when the parameters satisfy the following constraints:

$$\{\begin{array}{c} b_{P}w^{SP}w^{PS}-b_{P}b_{A}w^{SA}w^{AP}w^{PS}=1\\ b_{P}c_{A}w^{AP}w^{PS}=1\\ a_{P}=b_{P}a_{A}w^{AP}\\ w^{SA}=1 \end{array}$$ (32)

This analysis shows that even if the f-I curves in GPe have a generalized form described by Eqs 28 and 29, by setting the weights to the values satisfying the above conditions, the GPe can compute the function *STN*–log *STN*.

### Connections between GPe and striatum

The model presented so far describes the computations only in a subset of nuclei in the larger cortico-basal-ganglia network that, when embodied in Fig 1, does not include the connections between GPe and striatum. However, there are prominent projections from the striatum to the GPe [2,3], and arkypallidal neurons densely innervate the striatum [20]. We now discuss how these bidirectional connections can be included in the model.

**Fig 8:**
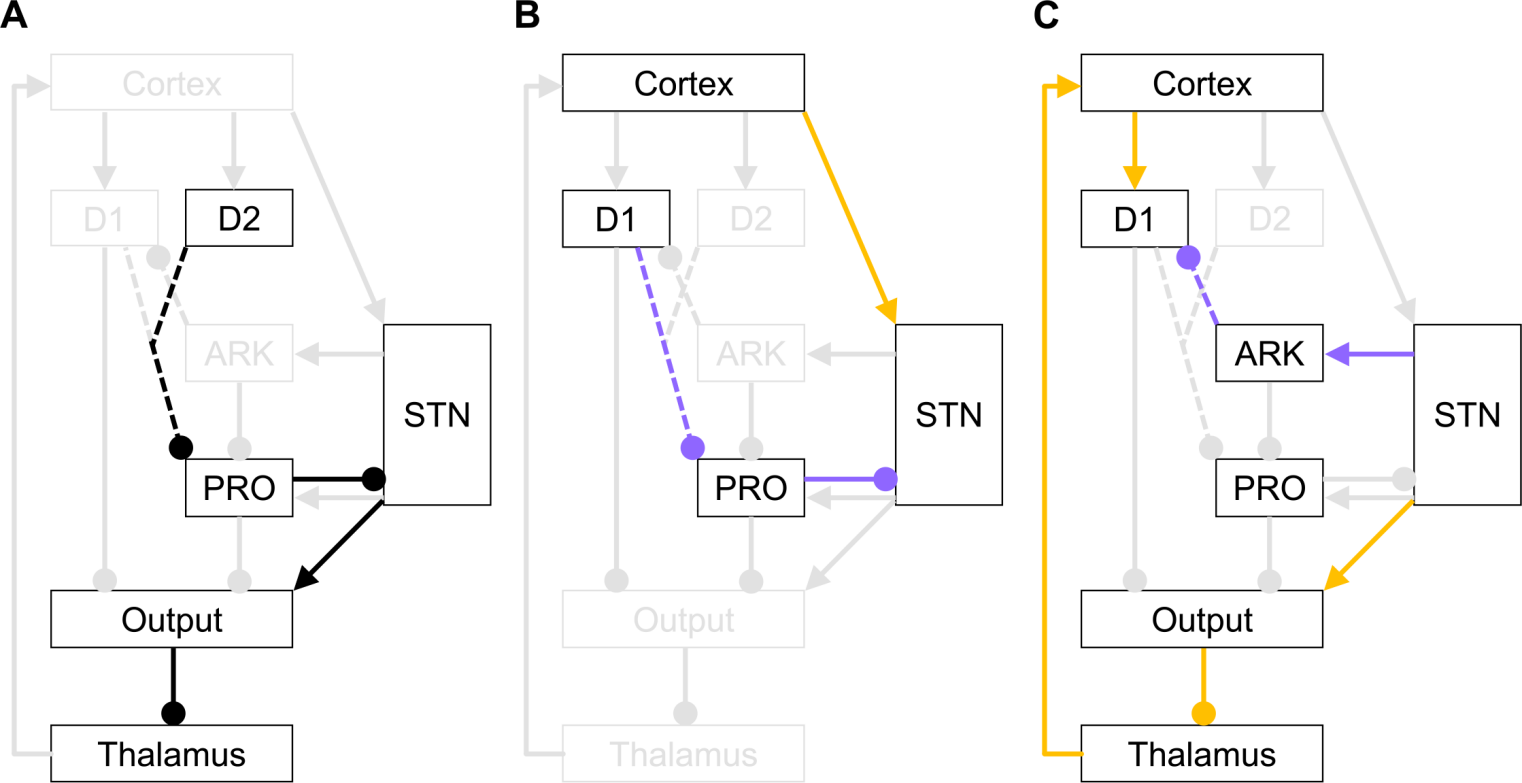
Connectivity between GPe and striatum. Rectangles denote neural populations: D1 and D2 label striatal neurons with D1 and D2 receptors, while PRO and ARK label prototypic and arkypallidal neurons respectively. Arrows and lines ending with circles denote excitatory and inhibitory connections respectively. Black and coloured parts of the circuit highlight the pathways illustrated in each panel. Purple pathways denote routes alternative to those shown in orange. The connections between striatum and GPe are dashed to indicate that it is not known which groups of neurons in striatum and GPe are interconnected–the figure shows only the hypothetical connections discussed in the main text. **(A)** Part of the indirect pathway connected with movement inhibition. **(B)** Routes providing evidence for actions to STN. **(C)** Routes providing normalization to the striatum.

The striatal projection neurons can be divided into those expressing type 1 dopamine receptors or type 2 dopamine receptors (D1 and D2), and they are thought to be involved in initiation and inhibition of actions, respectively. Let us first discuss the D2 striatal neurons for which GPe is the main target of their projections [3]. The connection from the D2 neurons to GPe is a critical part of the so-called indirect pathway (Fig 8A) involved in inhibiting the actions that in the past resulted in negative feedback [4]. Although there are no anatomical data that definitively describe to which GPe cell type(s) the D2 striatal neurons project, Fig 8A includes connections from D2 neurons to prototypic neurons, as only such connections would allow D2 striatal neurons to inhibit actions (if the D2 neurons projected to arkypallidal neurons, their activity would facilitate rather than inhibit action selection, because the pathway D2-ARK-PRO-STN-OUT-TH would include 4 inhibitory connections, so it would be effectively excitatory). The striatal D2 neurons are not included in the optimal action selection model (see Fig 1), because the MSPRT framework assumes that one of the actions is correct and should be taken on a given trial. By contrast, in certain situations, any action may lead to negative consequences, and the D2 striatal neurons are likely to ensure that no action is then taken. To include the D2 striatal neurons and their projections to GPe, the model has to be extended beyond the MSPRT framework, which is the subject of ongoing work.

It has been also reported that GPe receives input from most of the striatal neurons that target the output nuclei of the basal ganglia [42], i.e. the D1 striatal neurons. These striatal neurons are included in the model shown in Fig 1, but their projection to GPe is not. This pathway may form an alternative route by which the evidence for actions represented in cortex and striatum can be provided to STN. Please note that if the D1 striatal neurons project to prototypic neurons, the pathway CTX-D1-PRO-STN involves 2 inhibitory connections so it is effectively excitatory, similarly as the hyperdirect pathway CTX-STN (Fig 8B). Note that this would not be the case if striatal D1 neurons projected to arkypallidal neurons, as connection D1-ARK-PRO-STN would be effectively inhibitory, thus for simplicity we only consider the connections from striatal D1 neurons to prototypic GPe neurons.

We now show that adding the connection from striatal D1 neurons to prototypic GPe neurons, while also appropriately reducing the connection from cortex to STN, would not change the activity in the STN in the model, and hence the model would still implement optimal action selection.

Let us consider a modified version of the model in which we allow arbitrary weights of connections from cortex to STN, which we denote by *w*^*CS*^, and from striatal D1 neurons to prototypic neurons, which we denote by *w*^*XP*^. Since we assume that the activity of striatal D1 neurons reflects the cortical inputs, the activity of STN and GPe populations in this model becomes:

$$STN_{k}=\exp\left(w^{CS}CTX_{k}-w^{PS}PRO_{k}\right)$$ (33)

$$PRO_{k}=f_{P}\left(w^{SP}STN-w^{AP}ARK_{k}-w^{XP}CTX_{k}\right)$$ (34)

$$ARK_{k}=f_{A}\left(w^{SA}STN\right)$$ (35)

The computation in this modified model and the one described in the Models Section will remain the same if the activity in STN described in Eq 33 is the same as that described by Eq 16. Equating the arguments of exponentials in the two equations, one obtains the following condition:

$$w^{CS}CTX_{k}-w^{PS}PRO_{k}=CTX_{k}-STN+\log STN$$ (36)

Substituting Eqs 34 and 35, 28 and 29, and the conditions of Eq 32, we get:

$$\left(w^{CS}+w^{XP}b_{P}w^{PS}\right)CTX_{k}-STN+\log STN=CTX_{k}-STN+\log STN$$ (37)

It is evident that the above condition is satisfied when:

$$w^{CS}+b_{P}w^{XP}w^{PS}=1$$ (38)

Thus, if the GPe neurons respond to their inputs as described by Eqs 28 and 29, the computations in the model do not change if a portion of cortical input is delivered to the prototypic neurons (via striatum) rather than to STN.

What could be the benefit of the existence of two alternative routes by which the evidence for actions can be provided to the STN? The version of the model presented in this paper describes decision making in highly practiced tasks. It assumes that the mapping between stimuli and actions is already consolidated in cortico-cortical connections, and that information from sensory cortex is integrated with prior evidence in the frontal cortex (see Fig 1). Thus the direct connection from frontal cortex to STN is the fastest route to provide the integrated evidence for actions to STN. However, experimental data [43] suggest that while acquiring a task the stimulus-response mapping is initially learnt in striatum, and many computational models of this learning process have been proposed [4,44]. Consequently, a version of the optimal action selection model describing newly learnt tasks [10] assumes that the information brought by stimulus is integrated with prior evidence in the striatum. Thus in this case the connection D1-PRO-STN is a fast route to provide integrated evidence to STN (otherwise it would have to go via D1-OUT-TH-CTX-STN). Thus in summary, the two alternative routes to STN could allow fast information transfer to STN in different phases of task acquisition.

It has been also shown that arkypallidal neurons innervate striatum to a much larger extent than prototypic neurons [20]. Moreover, arkypallidal neurons are the only type of GPe neuron that has been shown so far to innervate striatal projection neurons [20]. This connection could provide a quick route for the STN feedback to reach the striatum (Fig 8C) and thus normalize the probabilities of actions represented in striatum. Without this connection, the feedback from STN would need to take a long route via thalamus and cortex (Fig 8C). We show below that adding the connection from arkypallidal neurons to striatal D1 neurons and appropriately reducing the connections from arkypallidal neurons to STN (via prototypic neurons) would not change the activity in the output nuclei in the model, and hence the model would still implement optimal action selection (it is not known whether the arkypallidal neurons project to D1 and/or D2 striatal neurons, but we only consider here the projections to D1 neurons, as the D2 neurons are not included in the model in the MSPRT framework–see above).

Let us consider a modified version of the model, in which we allow arbitrary strengths of connections from arkypallidal neurons to D1 striatal neurons, which we denote by *w*^*AX*^ (for simplicity we no longer consider connections from striatum to GPe). The activities of neural population in STN, GPe and output nuclei in this model become:

$$STN_{k}=\exp\left(CTX_{k}-w^{PS}PRO_{k}\right)$$ (39)

$$PRO_{k}=f_{P}\left(w^{SP}STN-w^{AP}ARK_{k}\right)$$ (40)

$$ARK_{k}=f_{A}\left(w^{SA}STN\right)$$ (41)

$$OUT_{k}=-CTX_{k}+w^{AX}ARK_{k}+STN$$ (42)

Let us now recall that in the original model in the Models Section, the activity in the output nuclei was the following function of cortical activity:

$$OUT_{k}=-CTX_{k}+\log\sum_{k=1}^{N}\exp CTX_{k}$$ (43)

In order for the two models to select action in the same way, they need to have the same levels of activity in the output nuclei, thus comparing Eqs 42 and 43 we obtain the following condition:

$$w^{AX}ARK_{k}+STN=\log\sum_{k=1}^{N}\exp CTX_{k}$$ (44)

We will now compute how the right hand side of Eq 44 depends on parameters of GPe neurons in the modified model. Substituting Eqs 40 and 41 into 39, we obtain:

$$STN_{k}=\exp\left(CTX_{k}-w^{PS}f_{P}\left(w^{SP}STN-w^{AP}f_{A}\left(w^{SA}STN\right)\right)\right)$$ (45)

Summing over alternatives, taking logarithm and re-arranging, we obtain:

$$\log\sum_{k=1}^{N}\exp CTX_{k}=\log STN+w^{PS}f_{P}\left(w^{SP}STN-w^{AP}f_{A}\left(w^{SA}STN\right)\right)$$ (46)

Substituting Eqs 46 into 44 and rearranging terms we obtain a general condition that needs to be satisfied for the modified model to be equivalent to that in the Model Section:

$$STN-\log STN=w^{PS}f_{P}\left(w^{SP}STN-w^{AP}f_{A}\left(w^{SA}STN\right)\right)-w^{AX}f_{A}\left(w^{SA}STN\right)$$ (47)

Substituting Eqs 28 and 29, we observe that the above condition becomes satisfied when:

$$\{\begin{array}{c} b_{P}w^{PS}w^{SP}-b_{P}b_{A}w^{PS}w^{SA}w^{AP}-b_{A}w^{SA}w^{AX}=1\\ b_{P}c_{A}w^{AP}w^{PS}+c_{A}w^{AX}=1\\ a_{P}w^{PS}=b_{P}a_{A}w^{AP}w^{PS}+a_{A}w^{AX}\\ w^{SA}=1 \end{array}$$ (48)

The above conditions are generalizations of those in Eq 32. In particular, the second condition implies, that the computations in the model will not change if a proportion of output from arkypallidal neurons is sent to striatum instead of to STN via the prototypic neurons. This rebalancing of the network does not change the computation performed by the model, but could stabilize the dynamics in the striatum-GPe network (as pathway D1-PRO-STN-ARK-D1 is effectively inhibitory so forms a negative feedback loop).

## Discussion

This paper presents a model of the microcircuits within STN-GPe elucidating how this network could compute the logarithm of the normalization term in Bayes’ theorem that underpins the procedure for optimal action selection. The model is based on recently reported connectivity of two types of GPe neurons, and the shapes of their f-I curves. Our results suggest that GPe neurons have a diversity of structural and electrophysiological properties necessary for contributing to optimal action selection in the cortico-basal-ganglia circuit.

In the optimal action selection model, the input from the STN to the output nuclei plays a very similar function as in other models of action selection [45,46]. In particular, the surround inhibition model [45] assumes that when one of the actions is selected, the input from the STN ensures that others are inhibited. In the optimal action selection model, the input from STN normalizes the neural representation of probabilities so that they add up to 1. Hence it ensures that if estimated probability of one action increases, the probabilities of other action decrease (as in [45]). The conflict model [46] proposes that when neural populations selective for two different actions are both active, the input from the STN postpones the action initiation. In the optimal action selection model, the input from the STN ensures that if neurons representing two actions both receive equally high inputs, the probabilities of these two actions will not exceed 0.5, thus none of the actions will be initiated until the conflict is resolved (as in [46]). Thus the input from the STN in the optimal action selection model fulfils the roles assigned to it by previous theories, but the model additionally proposes that this input is a particular function of cortical activity (Eq 7) which allows optimal action selection. To compute this function the STN needs to interact with the neurons in the GPe.

### Interpretation of the results

The range of values of the weights in Fig 6B should not be treated as a precise prediction on the connectivity of GPe neurons, mostly because other key inputs to GPe neurons (e.g. from striatum and thalamus) were not considered to avoid introducing additional free parameters in the optimization procedure. Some of the fitted weights in Fig 6B did not match previous estimates of the effective connectivity of the STN-GPe network in Parkinsonism [36] (the difference occurred in the connection weights between the two types of GPe neuron as well as from STN to GPe). However, please note that the two sets of weights were based on different fitting procedures applied to different data sets recorded from different animals in different labs, and most importantly, *in vitro* data of neurons from dopamine-intact animals were used here, while *in vivo* data from animal models of Parkinson’s disease were used in the previous study [36]. Dopamine depletion may change the connectivity of prototypic and arkypallidal neurons, as it changes the relationship between their *in vivo* firing rates, such that the prototypic neurons, which in intact animals are more active than arkypallidal [18,19], become less active [19,21]. This parallels the differences in estimated connectivity from STN to prototypic and arkypallidal neurons, as in the present study both populations were estimated to receive similar excitation from STN (Fig 6B) whereas, in the previous study of the Parkinsonian STN-GPe network, prototypic neurons were estimated to receive less input from STN than arkypallidal neurons [36]. The previous estimates of effective connectivity [36] are consistent with our analysis of connections from striatum to GPe as they both suggest that prototypic neurons should receive more input from striatum than arkypallidal neurons.

While finding the connections of GPe neurons for which they compute function *STN*–log *STN*, we accounted for differences in the shapes of f-I curves for each neuron. Such diversity contributed to making the system high dimensional, thus enabling it to find multiple solutions to the optimisation problem. Contrary to our initial expectation, the different solutions did not exhibit a specific pattern of connectivity. Indeed, one might predict that linear prototypic neurons project to STN more strongly than logarithmic prototypic neurons project to STN. This was not the case, which highlights the flexibility and robustness of the network considered to find solutions that support optimal decision making.

Simulations investigating the dynamics of STN-GPe circuit (Fig 7) revealed that a circuit with realistic time constants and synaptic transmission delays converged to a state in which the activity of STN neurons encoded log *P*(*S*), except for high cortical input when the network produced sustained oscillations. Nevertheless, it has to be noted that weight parameters were estimated using a model without delays, so it is possible that other combinations of weights can be found for which the STN-GPe circuit performs the computation required for optimal action selection, but is more stable. Furthermore, even when the oscillations are present in Fig 7, the firing rate averaged over time is close to that required for the optimal action selection. It is also possible that log *P*(*S*) is encoded in the power of oscillations in STN in addition to the firing rate. The frequency of oscillations observed in Fig 7 is close to the beta range, and the beta oscillations are thought to be involved in inhibition of movement [47] and their power in STN is increased in Parkinson’s disease [48]. Given that the feedback from STN also slows down action initiation in the optimal action selection model (e.g. in the presence of conflict–see earlier Discussion), its representation in the power of beta oscillations would be consistent with the akinetic properties of these oscillations.

### Future directions

In this paper we have shown that the connections of GPe neurons required for optimal action selection can be found. However, we have not shown how these connections arise from a self-organization process employing local plasticity rules, in which the change in a synaptic weight depends on the activities of presynaptic neurons, postsynaptic neurons and levels of neuromodulators. Developing such plasticity rules would be an important direction for future work.

The model presented in this paper for simplicity assumed that each GPe neuron received input from all STN neurons. Although the projections from STN to GPe are widespread [27], single STN neurons do not contact all GPe neurons. It will be important to investigate in the future if a model with a more realistic pattern of connectivity between STN and GPe can also approximate optimal action selection.

So far the main experimental support for the optimal action selection model comes from *in vitro* studies of properties of isolated basal ganglia neurons: in the original paper introducing the model [9] it was already pointed out that the f-I curves of STN neurons have exponential shape for a wide range of firing rates, and here we show that the f-I curves of two types of GPe neuron can support the computation of function *STN*–log *STN*. To provide further support for the model, it is necessary to show that neurons embedded in the circuit *in vivo* also behave in the predicted way. For example, one could investigate how the activity of GPe neurons *in vivo* depends on the activity in STN (which could, for example, be parametrically manipulated using optogenetic actuators). The model predicts that the activity of prototypic GPe neurons should be a convex function of STN activity, proportional to *STN*–log *STN*, while the activity of arkypallidal neurons should be a concave function of STN activity. As far as we are aware, this prediction is specific to the model proposed in this paper, and not made by any other model of action selection in the basal ganglia.

A crucial test for the optimal action selection model would involve measuring whether the average activity in the STN during decision making encodes log *P*(*S*) (see Fig 1). For example, human participants could be asked to perform a task equivalent to that described at the start of Models section, and the STN activity after each cue could be measured either with high resolution fMRI or via deep brain stimulation electrodes, if the study were performed with Parkinson’s patients. In that case the model would predict higher amplitude of oscillations in STN after presenting stimuli with higher *P*(*S*), because the simulations in Fig 7 suggest that more oscillatory activity is produced with higher STN activity.

The framework of the model we present here could be employed in the future to investigate how the computations in the STN-GPe circuit change in cases of dysfunction, such as in Parkinson’s disease where the chronic loss of dopamine grossly disturbs circuit dynamics to impede the initiation and performance of actions. Our model emphasises that the f-I curve shapes and connection strengths of STN and GPe neurons are important for action selection. It is thus noteworthy that the shapes of STN neuron f-I curves depend on postsynaptic dopamine receptors [49], that the strengths of glutamatergic and GABAergic inputs to STN and GPe neurons are tuned by presynaptic dopamine receptors [50–52], and that the impact and structure of pallidosubthalamic connections are altered after dopamine depletion [53] as a result of homeostatic plasticity [54]. Our model also predicts that the autonomous firing rates of prototypic and arkypallidal neurons are necessarily different, with the former cell type exhibiting substantially higher rates of activity. With this in mind, it is notable that the autonomous firing of GPe neurons is grossly disturbed (and lost in a subset of neurons) in experimental Parkinsonism [55,56]. In conclusion, current evidence suggests that there are multiple ways by which dopamine loss in Parkinson’s disease could affect the input-output functions and connection strengths of STN and GPe neurons, such that, ultimately, prototypic GPe neurons might not efficiently compute the function *STN–*log *STN* that supports the optimal action selection.

## Supporting Information

S1 Table Firing rate of individual neurons as a function of injected current. (PDF)
