# Supplementary material for: Properties of Neurons in External Globus Pallidus Can Support Optimal Action Selection
Source: PLoS Comput Biol. 2016 Jul 7;12(7):e1005004. doi: 10.1371/journal.pcbi.1005004 (PMC4936724; doi:10.1371/journal.pcbi.1005004)
Supplement: S1 Table — (PDF) [file pcbi.1005004.s001.pdf]

Injected current (pA)	Firing rate (Hz)																																			
	Prototypic																		Arkypallidal																	
-70	0	0	0	0	0	0	0	0	0	0	0	0	0	0	0	0	0	0	0	0	0	0	0	0	0	0	0	0	0	0	0	0	0	0	0	0
-60	0	0	2	0	0	0	0	0	0	0	0	0	0	0	0	0	0	0	0	0	0	0	0	0	0	0	0	0	0	0	0	0	0	0	0	0
-50	0	0	9	0	0	0	0	0	0	0	0	0	0	0	0	0	0	0	0	0	0	0	0	0	0	0	0	0	0	0	0	0	0	0	0	0
-40	0	0	14	0	2	0	0	0	0	0	0	0	0	0	0	0	0	0	0	0	0	0	0	0	0	0	0	0	0	0	0	0	0	0	0	0
-30	0	0	18	6	2	0	12	0	0	0	0	4	0	2	0	0	0	0	0	0	0	0	0	0	0	0	0	0	0	0	0	0	0	0	0	0
-20	4	0	21	10	13	0	32	2	7	0	0	8	0	8	0	0	0	0	6	0	0	2	0	0	4	0	0	0	0	0	0	0	0	0	0	0
-10	18	2	29	18	17	6	49	6	14	0	8	10	2	10	10	8	0	6	10	0	4	4	4	0	6	2	0	0	0	0	4	0	0	0	0	2
0	26	4	31	21	26	14	53	11	22	6	23	13	14	12	23	13	8	5	19	3	9	8	6	1	9	1	2	3	2	9	5	5	1	2	1	6
25	44	9	38	32	38	24	81	19	34	17	46	19	33	24	42	25	15	9	31	15	27	12	12	10	16	7	14	9	9	15	10	14	8	10	7	14
50	56	14	48	42	48	30	100	26	44	29	63	23	46	32	56	36	21	13	40	24	40	17	17	15	20	11	24	14	15	23	15	22	12	17	12	19
75	68	18	55	52	57	35	115	33	53	37	77	27	58	38	68	44	27	18	49	31	51	22	22	21	24	15	33	20	19	28	19	31	16	24	15	25
100	78	22	65	60	67	40	128	38	62	46	85	31	68	43	81	50	32	21	56	37	61	28	27	27	29	19	39	26	23	34	23	40	21	29	19	29
125	87	27	73	67	74	46	136	43	69	52	95	35	78	49	91	57	36	25	60	42	69	32	30	32	31	22	47	31	26	40	28	48	24	33	23	33
150	133	30	83	72	82	51	142	47	77	56	112	38	87	52	103	62	41	29	67	45	77	36	34	39	33	25	52	35	28	48	29	57	29	37	27	37
175	99	33	90	78	89	56		52	82	60	114	40	96	55	112	67	44	32	71	48	83	41	38	44	35	27	58	38	29	56	33	66	33	42	30	40
200	109	36	95	83	95	62			89	61	121	43	106	57	121	72	48	34	75	51	90	45	41	50	38	30	61	42	30	59	35	74	36	46	33	42
225	114	39	102	88	100	69			95	65	130	46	114	59	130	77	51	37	80	54	97	49	44	55	39	32	64	46	30	67	37	84	41	49	35	46
250	121	40	107	92	108	71				67		48	122	63	137	78	54	40	83	56	103	52	46	60	41	34		49	31	74	41	91	45	52	38	48
275	126	43	110	97	111	76				69		51	131	64	146		57	42	86	59	109	55	48	65	42	35		49		80	43	98	51	55	40	50
300	133	44	112	101	115	79						53	137	67	149		59	45	89	60	117	58	50	70	43	36		56		88	46		55	58	41	53
325	146	45	110	103	124							55	144	69	153		61	47	92	64	123	59	53	76	44	38		59		95	48		58	59	43	55
350	187	47	121	101	126							57	150	69	161		63	49	96	66	130	63	53	81	45	40		64		106	50		59	62	45	56
375	167	48	126	97	139							59	155	72	166		65	51	93	69	137	65	54	86	46	41				111	54		62	65	47	58
400	206	49	129	111								60	161	72	170		66	53	93	69	147	67	55	91	47	41								67	48	60
425	255	49	128	109								62	165	75	171		68	56	91	73	150	68	56	97	47	42								68	50	62
450	518	51		103								63	170	78	173		68	57	97	76	156	69	58	103	48	43								71	51	64
475		52		92								65	176	79	176		70	59	99		163	69	58	106	48	44								73	52	
500		53		92								67	181	81	179		70	60	99		168	74	59	114	49	45								75	53	
525		54		92									186	84	181		71	63	100		169	88	59	121	49	45								77	54	
550		55		90									189	89			72	65	102		175	97	59	127	50	44								80	55	
575		56		106									191	92			72	68	104		181	96	59	133	49	49								83	56	
600		61											196	101			72	70			184	107			50									83	56	
